# A Mobile App to Enhance Behavioral Activation Treatment for Substance Use Disorder: App Design, Use, and Integration Into Treatment in the Context of a Randomized Controlled Trial

**DOI:** 10.2196/25749

**Published:** 2021-11-03

**Authors:** Catherine E Paquette, Dillon T Rubalcava, Yun Chen, Deepika Anand, Stacey B Daughters

**Affiliations:** 1 Department of Psychology and Neuroscience University of North Carolina at Chapel Hill Chapel Hill, NC United States; 2 CBT Center of Chicago Chicago, IL United States

**Keywords:** substance use disorder, smartphone app, mHealth, behavioral activation, mobile phone

## Abstract

**Background:**

Group-based formats typically used in low-resource substance use disorder (SUD) treatment settings result in little individual attention to help reinforce and guide skill use, which may contribute to poor posttreatment outcomes. Smartphone apps offer a convenient, user-friendly, and cost-effective tool that can extend the reach of effective SUD treatments. A smartphone app was developed and integrated into a group-based, brief behavioral activation (BA) treatment for SUD to increase engagement in treatment skills outside clinician-administered sessions.

**Objective:**

This study aims to describe the features of the app and its use and integration into treatment, report the participants’ self-reported feasibility and acceptability of the app, and discuss challenges and provide recommendations for future smartphone app integration into behavioral treatments for SUD.

**Methods:**

A total of 56 individuals recruited from intensive outpatient SUD treatment received a smartphone-enhanced BA treatment, the Life Enhancement Treatment for Substance Use. Self-reported weekly app use and reasons for nonuse were assessed at posttreatment and at 1- and 3-month follow-ups. In addition, 2-tailed *t* tests and chi-square tests compared the self-reported use of each app component and overall app use over time.

**Results:**

Participant feedback suggested that the integration of the smartphone app into the Life Enhancement Treatment for Substance Use was feasible and well accepted, and participants found the app useful for planning value-based activities outside of sessions. Self-reported app engagement decreased over the follow-up period: 72% (39/54) of participants reported using the app at posttreatment, decreasing to 69% (37/54) at the 1-month follow-up and 37% (20/54) at the 3-month follow-up. Participants reported forgetting to use the app as a primary reason for nonuse.

**Conclusions:**

This study provides support for the feasibility and acceptability of smartphone-enhanced BA treatment, offering promise for future research testing the integration of technology into SUD treatment. Design decisions may help streamline smartphone integration into treatment, for example, allowing participants to download the treatment app on their own phones or use a low-cost study smartphone (or offering both options). Long-term app engagement may be increased via built-in reminders, alerts, and in-app messages.

**Trial Registration:**

ClinicalTrials.gov NCT02707887; https://clinicaltrials.gov/ct2/show/study/NCT02707887

## Introduction

### Background

Limited access to evidence-based substance use disorder (SUD) treatment is a pervasive problem in the United States. Of the 20.3 million Americans who experienced an SUD in the past year, only approximately 12% received treatment at a specialty facility [1]. Treatment providers at these facilities often lack extensive training in evidence-based interventions [2]. Most SUD treatment is provided in a group-based format [3], in which participants receive little individual attention, and group-based peer support models such as 12-Step Facilitation are the primary method of care at many treatment centers [2,4]. Ongoing monitoring and aftercare for SUD treatment is rare [5,6], although prolonged treatment engagement is associated with better outcomes [7,8]. There is a clear need for evidence-based treatments that prioritize ongoing engagement and are accessible within low-resource SUD treatment settings (including treatment being provided by practitioners without graduate-level training).

### Behavioral Activation for Substance Use

The Life Enhancement Treatment for Substance Use (LETS ACT) [9] was developed to address this need. LETS ACT is a behavioral activation (BA) treatment, which aims to increase substance-free environmental reinforcement through the planning and execution of value-based activities. SUDs are characterized by reward deficits and a loss of drug-free positive reinforcement, resulting in decreased engagement in naturally rewarding (ie, drug-free) activities [10]. BA targets this lack of environmental reward by helping participants increase their daily engagement in activities that are enjoyable and important and thus provides opportunities for positive reinforcement. It has shown efficacy when delivered by individuals without professional training in psychotherapy [11], making it accessible for implementation in low-resource SUD treatment settings. LETS ACT, provided as a supplement to inpatient SUD treatment, has demonstrated effectiveness in reducing depressive symptoms [12] and rates of treatment dropout [13] as well as increasing rates of abstinence and decreasing substance-related consequences up to 1 year posttreatment [14].

Despite positive initial findings, there continues to be room to improve posttreatment outcomes by increasing out-of-session treatment engagement. Although significantly lower than a contact-matched control condition, more than 50% of LETS ACT participants reported using substances by 3 months posttreatment [14]. There is little individual attention to help reinforce and guide BA skills outside of the 6 group sessions. Research suggests that homework compliance is predictive of better treatment outcomes across psychotherapy modalities [15], including in cognitive behavioral treatments such as BA [16,17]. Thus, bolstering out-of-session engagement could be one way to improve posttreatment outcomes.

### Smartphone-Enhanced Treatment

Integrating smartphones into therapy is a promising strategy for increasing engagement outside group-based BA sessions. Features such as built-in guidance, prompts, and reminders can assist individuals in completing homework in a manner compliant with treatment guidelines, for example, by reminding participants to link planned activities to specific values. Users of smartphone apps for addiction recovery frequently cite the portability of apps as an advantage as well as their discreet nature [18]. Participants in BA treatments for depression have noted that smartphone-based treatments are more accessible to their everyday lives [19], which is key given the importance of daily activity planning in BA. Smartphone apps allow for quick access to skills learned in therapy within naturalistic settings, such as reviewing a plan for healthy coping behaviors in contexts with a high risk of substance use. Smartphone apps can also provide a cost-effective way of engaging patients [20], which makes them promising for low-resource, group-based SUD treatment settings.

Current research suggests that interventions involving smartphone apps are feasible and well accepted among individuals with SUDs. Research in SUD treatment samples demonstrates high rates of smartphone ownership and use, similar to the general population [21-23], and recent studies have found high overall acceptability of mobile health interventions for SUD [24]. Previous smartphone-based interventions have demonstrated positive effects on substance-related behavioral changes [24,25]. However, very few stand-alone apps use evidence-based interventions such as cognitive behavioral therapy [26], and research testing the integration of smartphone technology into established SUD treatments is lacking. Indeed, in a recent systematic review examining smartphone-based treatments for psychiatric diagnoses, only 2 of 27 studies identified by the review examined SUD interventions [27]. Of these, only 1 assessed a smartphone-enhanced treatment as opposed to a stand-alone app-based intervention, finding that the smartphone-enhanced treatment was associated with greater reductions in substance use [28]. Thus, there is a clear need for research examining the feasibility and utility of integrating smartphones into evidence-based SUD treatment.

### Study Objectives

This study reports feasibility data from a trial (NCT02707887) testing the effectiveness of a smartphone-enhanced BA treatment for SUD (smartphone-enhanced LETS ACT). The aims of the study are to (1) describe the features of the app and its use and integration into treatment, (2) report participants’ self-reported feasibility and acceptability of the app, and (3) discuss challenges and provide recommendations for future smartphone app integration into behavioral treatments for SUD.

## Methods

### Development of the LETS ACT App

The LETS ACT app was designed to largely reflect the paper treatment materials used in previous studies of LETS ACT, with a number of added features to facilitate theory-driven treatment engagement. In the development phase, the research team drew from prior research and consultation with researchers and clinicians with expertise in SUD treatment and the development of technology to enhance behavioral interventions. The design included app features intended to address some of the limitations of paper materials (eg, providing in-app suggestions for improving homework compliance based on the user’s weekly progress). Furthermore, the app was designed to collect daily mood and substance use data. The final app was developed through an iterative piloting process, which included testing a web-based version before piloting the app with individuals in inpatient SUD treatment.

### Design of This Study

The data presented here come from a single-site, 3-arm trial conducted at an intensive outpatient SUD treatment center in Raleigh, North Carolina, comparing smartphone-enhanced BA with standard BA and treatment as usual (TAU). The focus of this analysis is to determine the feasibility and acceptability of the smartphone-enhanced treatment condition before a future report of the main outcomes of the parent trial. All participants received TAU. A total of 65 participants were randomized to smartphone-enhanced LETS ACT and attended at least one session of treatment; of these, 56 attended a second session and received a smartphone. Data for this study were collected at the pretreatment assessment, posttreatment, and at 1- and 3-month posttreatment follow-ups (FU1 and FU3). All study procedures were approved by the institutional review board.

### Sample and Recruitment

Patients at the outpatient facility were primarily low-income individuals with a range of SUD diagnoses who voluntarily enrolled in the treatment. Patients were recruited by the research team weekly through announcements at the end of the TAU treatment groups and by approaching individuals after these groups were released. Interested individuals were assessed for eligibility, provided informed consent, and completed the pretreatment assessment. Randomization occurred at the group level using a computerized urn randomization program, and participants were blinded to the condition (ie, participants were recruited in waves and were unaware of the 3 arms of the trial). Study exclusion criteria were (1) age >65 or <18 years, (2) less than fifth grade English reading level (ie, score <42 on the Wide Range Achievement Test), (3) current impairment due to psychotic symptoms, (4) completion of >6 weeks of TAU, and (5) inability to give written informed consent to participate. Following treatment, participants completed FU assessments at the outpatient treatment facility or a public location with adequate privacy (eg, public library).

### Intervention

#### Smartphone-Enhanced LETS ACT

The smartphone app was developed as an adjunct to LETS ACT [9]. LETS-ACT-SE is provided in small groups of 6 or fewer participants twice weekly over 3 weeks (6 sessions total). Each session begins with a discussion of the treatment rationale, including describing the cycle of negative mood, urges, and maladaptive behaviors (eg, substance use) and eliciting participant examples of how this cycle is experienced. Participants learn that the goal of treatment is to break this cycle by engaging in healthy, rewarding behaviors. They are taught that when an individual regularly engages in activities that generate a sense of enjoyment or accomplishment (or both), they are less likely to have urges to use substances or engage in other maladaptive behaviors in response to difficult emotions. Following the treatment rationale, participants record daily activities and rate them on enjoyment and importance to identify patterns of inactivation and opportunities to increase positive reinforcement.

**Figure 1:**
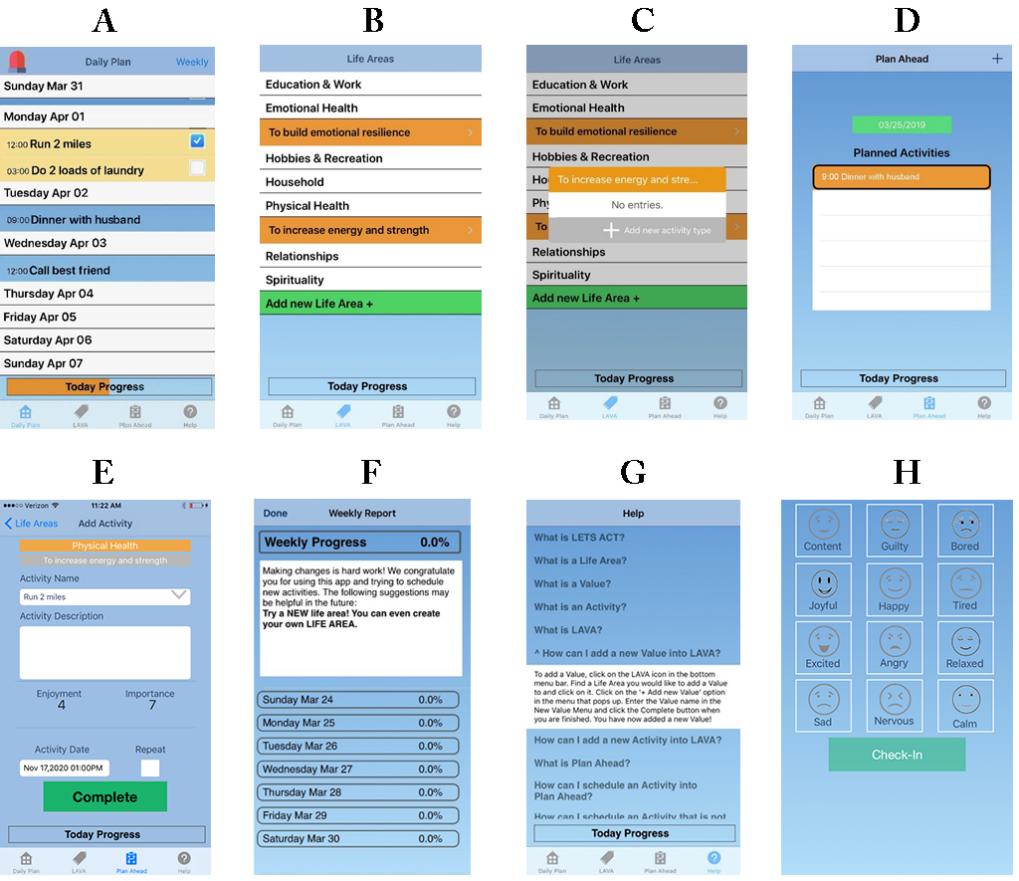
Screenshots of the LETS ACT app. A: Daily Plan with completed activity; B: Life Areas, Values, and Activities; C: Activity prompt in Life Areas, Values, and Activities feature; D: Plan Ahead; E: Rating enjoyment and importance; F: Weekly Progress; G: Help page; H: Mood ratings.

Next, emphasis shifts to an activity called Life Areas, Values, and Activities (LAVA). LAVA involves identifying activities associated with specific values and life areas (eg, education and work, emotional health, hobbies and recreation, and relationships). Participants are guided through the LAVA activity by selecting a life area that is important to them (eg, physical health), then identifying a value they hold related to that life area by answering the question, “What is important to me within this life area?” (eg, “It is important to me to increase energy and strength”). Participants then generate specific, measurable activities aligned with their values, with an emphasis on balancing enjoyable and important activities (eg, “In order to have energy and strength [value], I will walk in the park for 30 minutes [activity]”). Earlier sessions focus on tracking daily activities and creating LAVA lists. Later sessions shift focus to planning and implementing these activities in a daily plan (Figure 1A), problem-solving challenges to adherence, and posttreatment planning.

Participants are given home practice assignments after each session, which include instructions for the continued use of each component. For example, after the introduction of LAVA in session 2, participants are asked to record at least one value and activity for their chosen life areas. After the introduction of the daily plan in session 3, participants are asked to plan and complete at least one activity per day for the remainder of the treatment. Participants are encouraged to continue planning and completing activities after the completion of treatment using their smartphones; however, they are not given any specific assignments to complete during the FU period.

Participants in LETS ACT-SE were provided with Apple iPhone 6 smartphones with the LETS ACT app predownloaded during the second treatment session. Phone plans were set up and paid for by the research study; plans included unlimited calls and text messages and 4 GB of wireless data per month. The intent of this service was for participants to use their phone for regular use, thus allowing the research team to assess the feasibility of the app on a personal use device. This ensured that all participants had consistent access to the LETS ACT app (which was programmed specifically for the iPhone to limit development costs) as well as to reliable internet access throughout the study duration, allowing for ongoing data collection. At this time, they were given a brief introduction to the smartphones and LETS ACT app as well as a packet of information about basic features of the phones and instructions for use (eg, how to change settings). Participants absent in session 2 were given the phone and instructions at the next treatment session attended. Participants were introduced to each app component during the sessions, with a quick therapist-led tutorial followed by in-session practice. Participants were asked to use the smartphone app to record their homework. They were informed that the smartphones were theirs to use until their FU3 appointment, at which time they returned their phones to the research team and received monetary compensation. Individuals who lost their smartphone or forgot to bring it to treatment were provided with equivalent paper forms.

#### Treatment as Usual

All study participants were enrolled in a substance use disorder intensive outpatient program, in which treatment is based on the matrix model of intensive outpatient treatment [29]. The program included group therapy (average of 8-10 patients per group) for 3 hours per day, 3 days per week for 12 weeks, as well as weekly individual appointments with a case manager and up to 2 optional individual counseling sessions per week. Although group sessions do not have a set curriculum, they typically include individual check-ins, psychoeducation (eg, related to relapse prevention), and time to verbally process and share. Urine drug testing is implemented throughout the treatment, and positive drug tests are openly discussed within group sessions. Continued use of substances (aside from nicotine) is grounds for dismissal from the program.

### App Design and Components

Key components of the LETS ACT app include the LAVA library, Plan Ahead or Daily Plan, Weekly Progress, and Emergency button, accessible via icons on the home screen of the app (this is also the Plan Ahead screen; Figure 1A). Additional features include a Help page and a data collection mechanism for mood and substance use tracking.

#### Life Areas, Values, and Activities

The LAVA feature (Figure 1B) guides the user through the 3 steps of selecting value-based activities (described previously), reflecting the way the LAVA activity is taught during treatment. This was designed to increase the likelihood that the selected activities were value based. After selecting the LAVA icon, the user is presented with a list of life areas and an option to add a new life area. By tapping on a life area, the user is prompted to add a new value. Once complete, the value is listed in orange underneath the associated life area on the LAVA screen, and the participant can add activities by selecting the value (Figure 1C). Users can enter multiple values within each life area and perform multiple activities under each value.

#### Plan Ahead and Daily Plan

Planning value-based activities is central to the LETS ACT treatment, and the app includes 2 features that assist with this. The Plan Ahead feature (Figure 1D) allows the user to schedule value-based activities for specific days and times. By tapping a plus sign, the user is brought to the list of life areas, where they can either select an activity previously entered in the LAVA feature or enter a new activity (ie, by first selecting a life area, then entering a value and corresponding activity). Once an activity is selected, the user is prompted to rate the activity on enjoyment and importance (Figure 1E). Finally, the user can select a specific date and time to complete the activity, with the option of repeating the activity daily and/or weekly. The activity is entered into the user’s daily plan (Figure 1A), and upcoming planned activities are listed by date on the Plan Ahead screen (Figure 1D).

The Daily Plan feature is the home screen of the app (Figure 1A). Activities planned for the coming week are listed by day, and users can mark activities complete by checking a box at or after the assigned completion time (until midnight on the same day). Activities not marked complete by midnight are recorded as incomplete and removed from the Plan Ahead and Daily Plan screens.

#### Weekly Progress

On the home screen, an option in the top right corner allows the user to view their weekly progress, that is, the percentage of planned activities completed in the previous week (Figure 1F). An overall percentage is displayed at the top of the screen and the percentages for each day of the week underneath. In addition, this screen displays feedback and suggestions based on the user’s progress. The Daily Plan screen also features a *Today Progress* bar that fills in with orange based on the percentage of completed activities for the current day and turns green when 100% of activities have been completed (Figure 1A).

#### Emergency Button for High-risk Situations

The Emergency button appears as a red siren at the top left of the Daily Plan screen (Figure 1A) and allows the user to create a list of *emergency activities* or healthy coping behaviors they can use while experiencing difficult emotions and/or urges to use substances. The emergency screen lists the user’s emergency activities, which can be quickly added to the daily plan by selecting an activity title. Once selected, activities are marked complete at that specific date and time.

#### Help Icon

The Help icon brings the user to a page (Figure 1G) with a list of frequently asked questions and their answers, including information about the primary treatment components (eg, “What is a Value?”) and instructions for using the app features (eg, “How can I schedule an activity into Plan Ahead?”).

#### Research Functions

On opening the app for the first time each day, the user is prompted to rate their current mood (Figure 1H) and report any substance use (except for nicotine) since they last opened the app.

### Measures and Outcome Variables

A questionnaire administered at pretreatment assessed smartphone ownership, use, and likelihood of using a smartphone for a research study. In addition, *Diagnostic and Statistical Manual of Mental Disorders, Fifth Edition* (DSM-5) diagnoses for mood, anxiety, and SUD were assessed at pretreatment using the Mini-International Neuropsychiatric Interview [30]. Sociodemographic information (including age and education level) was assessed at all time points. Detailed information about the measures and outcome variables related to self-reported app use and app component feedback is provided in Table 1 and described below.

**Table 1:** Measures and outcome variables.

Construct	Variable description	Scale or possible value range	Time points
Past-week use of app components^a^	Multiple (all past week): 1. # days created new life areas, values, and activities using the LAVA^b^ library 2. # days scheduled ≥1 activity into daily plan using Plan Ahead 3. # days used the Emergency Button ≥1 time 4. # days viewed Weekly Progress 5. # days viewed Help icon	0-7 days	PT^c^
Average weekly use of app components in past month^a^	Multiple (all past month): 1. Average # days/week entered life areas, values, and activities using LAVA icon 2. Average # days/week used Daily Plan icon 3. Average # activities planned >1 week in advance using Plan Ahead icon 4. Average # days/week used Emergency button 5. Average # days/week viewed Weekly Progress 6. Average # days/week viewed Help page	0-7 days	FU1^d^ and FU3^e^
Any app use^a^	Whether participant reported using any (ie, one or more) app component at least 1 day per week since the previous assessment	Yes or no	PT, FU1, FU3
App component usefulness^f^	Degree to which participant agrees each component was a useful part of treatment: 1. LAVA library 2. Plan Ahead 3. Emergency 4. Weekly Progress 5. Help	Scale of 1 (strongly disagree) to 5 (strongly agree)	PT
Reasons for not using specific app components^f^	Reasons for not using: LAVA library Plan Ahead Emergency Weekly Progress Help	Can select all that apply from a list of reasons, select *other*, or indicate that it does not apply because the participant did use that component	PT
Reasons for low weekly app use^f^	Reasons for not using the app at least 3 times a week	Can select all that apply from a list of reasons, select *other*, or indicate that it does not apply because the participant did use the app at least three times per week	PT

^a^Described under Self-reported Use of App Components section.

^b^LAVA: Life Areas, Values, and Activities.

^c^PT: posttreatment.

^d^FU1: 1-month follow-up.

^e^FU2: 3-month follow-up.

^f^Described under App Component Usefulness and Reasons for Not Using section.

### Self-reported Use of App Components

A questionnaire administered at posttreatment assessed participants’ self-reported app engagement during the past week. Participants indicated the number of days in the past week that they used each treatment component outside of the treatment sessions. At FU assessments (ie, FU1 and FU3), participants were given a similar questionnaire that assessed engagement with the app components during the past month. This included the average number of days per week that the participant used each component of the LETS ACT app and details about their use (eg, the number of activities scheduled and completed and the number of days per week with at least one scheduled activity; Table 1). A dichotomous variable representing any app use at each time point was calculated, such that participants were coded as having used the app at each time point if they reported using one or more app components on 1 or more days per week since the previous assessment.

### App Component Usefulness and Reasons for Not Using

A questionnaire administered at posttreatment assessed participant feedback about the treatment and its components (Table 1). For each component, participants rated the degree to which they agreed that the component was a useful part of the treatment on a scale of 1 (strongly disagree) to 5 (strongly agree). The measure also included questions assessing the reasons for *not* using each component. Participants could choose any applicable reasons from a list (eg, did not remember to use the feature, did not think it would be helpful, and difficult to understand how to use it), record their own reason under *other*, or indicate that the question did not apply to them because they did use that component. An additional question inquired as to any reasons participants did not use the app at least three times per week. Similarly, participants could select from a list of reasons, select *other*, or indicate that it did not apply because they did use the app at least three times per week. Finally, the questionnaire included an open-ended question eliciting feedback about the smartphone-enhanced treatment overall, including the degree to which it was useful and any suggestions for improvement.

### Statistical Analyses

Data were analyzed using SPSS (version 25.0, IBM Corp). First, descriptive statistics were calculated for all variables used in subsequent statistical analyses. This included means, SDs, and ranges for continuous variables and percentages for all categorical variables. To examine the feasibility and acceptability of the LETS ACT app, summary statistics (eg, mean, median, and SD) were calculated to characterize participant ratings regarding the usefulness of each app component, as well as self-reported engagement with each component (ie, past-week use of app components at posttreatment and average weekly use of app components in the past month at FU1 and FU3). Chi-square tests were used to examine differences in the proportions of participants who reported any app use at each time point. Two-tailed paired-sample *t* tests were used to examine the differences in the mean ratings for app usefulness across components. The reasons for not using each app component and reasons for low weekly use of the app were also summarized.

## Results

### Sample Characteristics

Of the 56 participants who received a smartphone, 21 (38%) were women. Overall, 61% (34/56) of participants were White and 38% (21/56) were Black. The average age was 42.4 (SD 10.5; range 24-62) years, and the participants had an average of 12.1 (SD 3.0; range 1-21) years of education. In terms of substance use, participants reported an average of 3.8 (SD 6.9; range 0-30) days of substance use in the past 30 days at pretreatment. For DSM-5 SUDs, 73% (41/56) of participants met the criteria for alcohol use disorder, 59% (33/56) met the criteria for cocaine use disorder, 45% (25/56) met the criteria for opioid use disorder, and 30% (17/56) met the criteria for cannabis use disorder. Regarding psychiatric comorbidity at pretreatment, 11% (6/56) of participants met the DSM-5 criteria for a current major depressive episode, and the average score on the Beck Depression Inventory at pretreatment was 12 (SD 11.3; range 0-51), reflecting minimal depressive symptoms. A total of 29% (16/56) of participants met the criteria for at least one DSM-5 anxiety disorder, including 18% (10/56) for social anxiety disorder, 11% (6/56) for agoraphobia and generalized anxiety disorder, 9% (5/56) for panic disorder, and 7% (4/56) for obsessive-compulsive disorder. Furthermore, 11% (6/56) met the criteria for bipolar I disorder, and 9% (5/56) met the criteria for posttraumatic stress disorder.

### Smartphone Interruptions

Of the 56 participants who received a smartphone, 54 (96%) were retained in the study through the 3-month FU assessment. One participant withdrew from the study at posttreatment because of having a busy work schedule, and a second died (of non–study-related causes) between the posttreatment and 1-month FU assessments. Overall, 27% (15/56) of participants reported at least one interruption in their ability to use their smartphone during or after treatment up to the 3-month FU, including phone lost or stolen (n=8), inability to access phones due to incarceration (n=3), and other issues (eg, technical issues with the phone; n=4).

### Smartphone Ownership and Use at Pretreatment

Among participants who received a smartphone, pretreatment data from those who reported their current smartphone ownership (n=38) indicated that 79% (30/38) owned a smartphone they could use daily. In total, 14% (5/37) reported that they owned an iPhone, whereas 62% (23/37) reported having an Android phone (9/37, 24%, either provided an invalid response or reported that they did not know what type of phone they owned; the remaining participant had missing data). Overall, 76% (29/38) of participants reported that they used the internet and apps on their phones. Participants were asked how likely they would be to use a smartphone if one was provided for treatment on a scale of 1 (“I would never use it”) to 10 (“I would definitely use it”); the average rating was 8.42 (SD 2.82).

### Self-reported App Engagement

**Figure 2:**
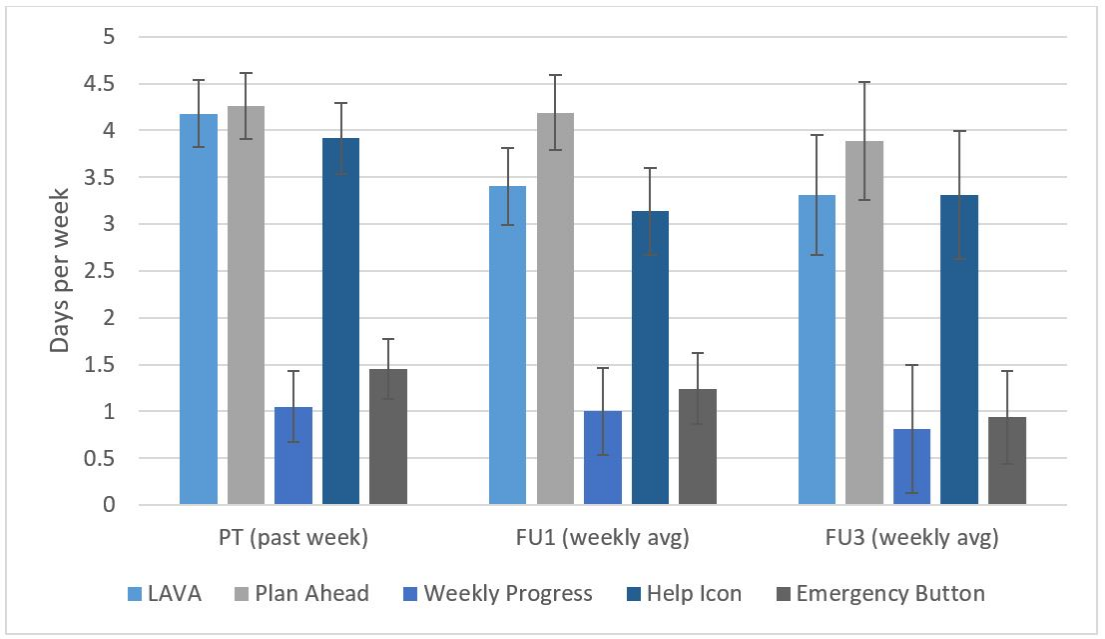
Weekly app use by component. FU: follow-up; LAVA: Life Areas, Values, and Activities; PT: posttreatment.

Self-reported app use data were obtained at posttreatment and FU assessments for 96% (54/56) of participants. Of these, 72% (39/54) reported any app use at posttreatment, 69% (37/54) reported any app use at FU1, and 37% (20/54) reported any app use at FU3. Chi-square tests indicated that the proportion of participants reporting any app use at posttreatment was significantly greater than the proportion reporting app use at FU3 (χ^2^_2_≥20.3; *P*<.001); significant decreases were also identified between FU1 and FU3 (*χ*^2^_2_≥11.5; *P*=.001). Considering only those participants who reported app use at each time point, 54% (21/39) reported using the app at least three times per week at posttreatment. Of those who used the app fewer than 3 times per week, common reasons included forgetting (10/18, 56%) and that it was difficult to use (4/18, 22%). Participants with any app use at posttreatment reported using the LAVA library, Plan Ahead, and Weekly Progress features an average of 4.18 (SD 2.19), 4.26 (SD 2.19), and 3.92 (SD 2.37) days per week, respectively; by the 3-month FU (FU3), participants reported using these features 3.31 (SD 2.57), 3.89 (SD 2.68), and 3.31 (SD 2.73) days per week on average (Figure 2). The Emergency button was used 1.05 (SD 1.88) and 0.81 (SD 1.80) days per week at posttreatment and FU3, respectively, and the Help page was used 1.44 (SD 1.98) and 0.94 (SD 1.98) days per week at posttreatment and FU3, respectively.

### App Component Usefulness

For each app component, participants rated their agreement with the statement that the app component was *a useful part of treatment* on a scale of 1 (strongly disagree) to 5 (strongly agree). The average ratings across all app components indicated that participants generally agreed that each component was useful (LAVA library: mean 4.39, SD 0.72; Plan Ahead: mean 4.33, SD 0.63; Emergency button: mean 3.97, SD 1.00; Weekly Progress: mean 4.22, SD 0.89; Help icon mean: 3.94, SD 0.87). Two-tailed paired *t* tests comparing usefulness ratings between components indicated that ratings for the LAVA library were significantly higher on average than the Emergency button (t_40_=2.56, SE 0.13; *P*=.01) and Help Page (t_40_=3.59, SE 0.87; *P*=.001), and ratings for the Plan Ahead feature were significantly higher than the Help Page (t_39_=2.69, SE 0.76; *P*=.01).

Participants were also asked to provide open-ended feedback about the smartphone-enhanced treatment, including suggestions for improvement. Of the 51 qualitative responses, 73% (37) expressed purely positive feedback. Written comments cited the usefulness of the treatment and highlighted the novelty of the smartphone-enhanced intervention and its utility in facilitating activity planning, helping participants to “be consistent”, and supporting their recovery. Positive comments included, “It was a good way to get me thinking about how my activities affect my emotions,” “[The app] keeps me on track to be more consistent on a daily basis with responsibilities,” and “It gave me more recovery tools to work with.” Suggestions for improvement included increasing overall ease of use, “more scheduling options” for activities, and providing a smartphone for use after the study FU period.

### Reasons for Not Using App Components

Across all components except for the Help page, forgetting to use the app component was by far the most frequently endorsed reason for lack of use (activity scheduling: 19/56, 34%; LAVA: 10/56, 18%; Emergency: 9/56, 16%; and Weekly Progress: 10/56, 18%), whereas not having the smartphone when the participant needed to use the app was generally the second-most endorsed (activity scheduling: 5/56, 9%; LAVA: 6/56, 11%; Emergency: 5/56, 9%; and Weekly Progress: 5/56, 9%). For the Help page, the most frequently endorsed reason was lack of need for the feature (19/56, 34%), whereas forgetting and not having a smartphone were the second-most endorsed reasons (3/56 each, 5%). To provide an example of the distribution of responses for 1 main component, Table 2 shows the reasons endorsed by participants for not scheduling activities.

**Table 2:** Reasons for not scheduling activities (n=56).

If there were days when you did NOT have an activity scheduled, it was because: (check all that apply)		Frequency, n (%)
This does not apply to me because I scheduled an activity on most days.		18 (32)
I did not remember to use the Daily Plan.		19 (34)
I did not have the smartphone with me when I needed to fill it out.		5 (9)
Filling it out took too much time or effort.		3 (5)
I had technical difficulties with the smartphone.		3 (5)
I did not think it would be helpful to me or my treatment goals.		1 (2)
Filling it out made me uncomfortable.		1 (2)
It was difficult to understand how to use it.		2 (4)
**Other**		6 (11)
	Lost or do not have phone	1 (2)
	Incarcerated or hospitalized	2 (4)
	Other or undisclosed	3 (5)
Missing or no response		13 (23)

## Discussion

### Self-reported App Use

With regard to self-reported app use, the majority (37/54, 69%) of participants reported continuing to use their LETS ACT app until 1 month posttreatment, but this proportion decreased significantly (to 20/54, 37%) by 3 months posttreatment. Research suggests that it is typical for mobile app use to quickly drop off. Indeed, many app users use an app only once; on average, user retention is 42% after 1 month and 27% after 3 months [31]. It appears, then, that the LETS ACT app follows a similar rate of decline in use to apps more broadly, although the level of retention was higher than average at both 1 and 3 months posttreatment. This level of attrition and lower continual engagement follows typical patterns of app use in mobile health interventions for SUD [32,33].

Regarding specific app components, participants generally agreed that each app component was a useful part of their treatment. The LAVA library and Plan Ahead feature, which were both essential to the core homework of activity planning, were the most used components and were also rated as more useful compared with features such as the Emergency button and Help page.

### Challenges and Recommendations Regarding Integration of Smartphone Technology

In this study, when participants did not use their apps or the individual app components, they generally reported that this was because of either forgetting or not having the smartphone with them. Both reasons for nonuse may reflect the drawbacks of giving participants a study smartphone rather than downloading the app on their own phones. This highlights a critical decision in smartphone-enhanced intervention research, that is, whether to provide smartphones for each participant to ensure consistent phone access or to offer an app that participants can download on their own phones to prioritize utility, ease of use, and generalizability. Research suggests that low-income people who use drugs have high rates of smartphone ownership, but that they tend to cycle through smartphones and have inconsistent access to wireless data [23], which presents a challenge for assessing the effectiveness of a smartphone-enhanced intervention. This study opted to provide smartphones to participants but found that participants often forgot to use them, which may be in part because they were not using the study phone as their primary phone. Giving participants the option to *either* download the app on their own phone *or* use a study smartphone may ultimately be ideal, although it requires additional resources (ie, developing app versions for both Android and iOS smartphones).

Forgetting to use the app was very common, so future studies examining the integration of smartphone apps into treatment may consider a range of strategies to mitigate this. This could include adding app features that target engagement. For example, reminders in the form of push notifications have been shown to significantly increase app use in smartphone interventions [34,35]. In-app direct messaging may also have the potential to increase engagement, as it provides a means for patients to communicate directly with treatment providers. As an additional strategy, therapists in smartphone-enhanced interventions could use time during treatment sessions to specifically target out-of-session app engagement with the ultimate goal of increasing homework compliance. For example, therapists could help participants make a plan to open their treatment apps at a regular time each day and to link this behavior to other daily activities (eg, “when I get out of bed each morning, I will open my app to see what activities I have planned for the day”).

Participants reporting that they did not have their smartphones with them when needed may reflect challenges inherent to maintaining treatment engagement among low-income people with SUD, many of whom experience significant instability in their daily lives. Some participants were incarcerated during the FU period, whereas others had smartphones that were lost or stolen; still others reported in informal conversations with research staff that they were nervous about losing their smartphones and chose to store them in a safe place, making them inconvenient to use regularly. Future studies with this population may wish to purchase low-cost smartphones (typically Android devices), which would increase the feasibility of offering replacement devices when needed. Given that this study found that most participants owned Android devices (vs iPhones [Apple Inc]) at pretreatment, they may have the benefit of being familiar to participants in addition to being more affordable.

The results of this study must be interpreted in the context of its limitations. Reliance on self-reported measures of app engagement may be associated with recall bias. In addition, the study sample was recruited from an intensive outpatient treatment center serving a primarily low-income, high school–educated clientele, and participation in the current intervention was offered in addition to standard treatment; the results may not be generalizable to other populations or treatment settings.

### Conclusions

There is a clear need for evidence-based SUD treatments that can be delivered at a low cost, and it is essential to find new and effective ways to engage participants in these treatments. Despite notable growth in the area of app-based psychological interventions, very little research has examined the impact of introducing smartphones into in-person therapy, especially in the area of SUD treatment. This study found evidence that integrating a smartphone app into a BA treatment for SUD is feasible and well accepted and that participants found the app useful for planning value-based activities, which is the core task of BA. However, the study also found that engagement with the app decreased over the FU period and that participants frequently reported forgetting to use the app, highlighting the need for further efforts to sustain out-of-session engagement over time. These findings must be interpreted in light of the specific study methods (eg, the provision of study smartphones to all participants); future research is needed to examine differences in app use when allowing participants to download treatment apps on their own smartphones. Studies are also needed to examine specific treatment contexts and participant characteristics that may be associated with receiving more benefit from smartphone-enhanced interventions.

## Abbreviations

BA: behavioral activation
*DSM-5*: *Diagnostic and Statistical Manual of Mental Disorders, Fifth Edition*
FU: follow-up
LAVA: Life Areas, Values, and Activities
LETS ACT: Life Enhancement Treatment for Substance Use
SUD: substance use disorder
TAU: treatment as usual
